# ONECUT2 reprograms neuroendocrine fate and is an actionable therapeutic target in small cell lung cancer

**DOI:** 10.1186/s10020-025-01267-6

**Published:** 2025-06-11

**Authors:** Mirian Gutiérrez, Irene Zamora, Raquel Iriarte, María José Pajares, Qian Yang, Chen Qian, Nerea Otegui, Joaquín Fernández-Irigoyen, Enrique Santamaría, Nicolas Alcala, Alexandra Sexton-Oates, Lynnette Fernández-Cuesta, Miguel Barajas, Alfonso Calvo, Luis M. Montuenga, Beatrice Knudsen, Sungyong You, Michael R. Freeman, Ignacio Encío, Mirja Rotinen

**Affiliations:** 1 Department of Health Sciences, Public University of Navarre, Pamplona, Navarre Spain; 2 Navarre Institute for Health Research (IdiSNA), Pamplona, Navarre Spain; 3 Department of Urology and Biomedical Sciences, Cedars-Sinai Medical Center, Los Angeles, CA USA; 4 Department of Computational Biomedicine, Cedars-Sinai Medical Center, Los Angeles, CA USA; 5 Center for Applied Medical Research (CIMA), University of Navarra, Pamplona, Navarre Spain; 6 Proteomics Unit, Clinical Neuroproteomics Laboratory, Navarrabiomed, Hospital Universitario de Navarra (HUN), Pamplona, Navarre Spain; 7 Rare Cancers Genomics Team (RCG), Genomic Epidemiology Branch (GEM), International Agency for Research On Cancer/World Health Organization (IARC/WHO), Lyon, France; 8 Department of Pathology, Anatomy and Physiology, Schools of Medicine and Sciences, University of Navarra, Pamplona, Navarre Spain; 9 CIBERONC, Madrid, Spain; 10 Department of Pathology, University of Utah, Salt Lake City, UT USA

**Keywords:** ONECUT2, SCLC, Tumor heterogeneity, Phenotypic plasticity, Therapeutic target

## Abstract

Small cell lung cancer (SCLC) is a highly aggressive malignancy with extremely poor prognosis. SCLC cells exhibit high plasticity and can progress from neuroendocrine (NE) to non-NE phenotypes. This dynamic evolution promotes treatment resistance and relapses, representing a challenge for targeted therapies in this elusive disease. Here we identify the transcription factor ONECUT2 (OC2) as a driver of plasticity in SCLC, leading to non-NE transcriptional states. OC2 is highly expressed in SCLC tumors compared to normal lung tissue and its expression is associated with heightened clinical stage and lymph node metastasis. We show that OC2 is a repressor of ASCL1, the NE master regulator transcription factor. In addition, OC2 upregulates non-NE programs through activation of c-MYC and Notch signaling. We also demonstrate that OC2 is required for growth and survival of SCLC cells and that it can be targeted with a small molecule inhibitor that acts synergistically with the standard combination of cisplatin and etoposide, providing a novel therapeutic strategy for OC2 active SCLC tumors.

**Supplementary Information:**

The online version contains supplementary material available at 10.1186/s10020-025-01267-6.

## Introduction

Small cell lung cancer (SCLC) has a dismal survival rate (Kalemkerian et al. 2013). Patients are often diagnosed with advanced stage disease, typically develop resistance to therapy within a year of diagnosis and die of metastases (Rudin et al. 2021). Traditionally perceived as a homogeneous entity by microscopic examination, recent molecular research suggests a highly heterogeneous cancer type. The proposed molecular subtypes ASCL1 (achaete-scute homolog 1), NEUROD1 (neurogenic differentiation factor 1), YAP1 (yes1-associated protein 1), and POU2F3 (POU class 2 homeobox 3) have unveiled distinct therapeutic vulnerabilities (Schwendenwein et al. 2021; Rudin et al. 2019). However, these molecular categories, also named SCLC-A, SCLC-N, SCLC-P and SCLC-Y, have sparked controversy, as the exclusive expression of YAP1 has been inconsistent (Redin et al. 2024; Baine et al. 2020; Qu et al. 2022). Another classification approach led to the proposal of SCLC-I subtype (Gay et al. 2021), an inflamed subtype characterized by a T-cell-inflamed gene expression profile, heightened immune cell infiltration and mesenchymal differentiation, that shows an enhanced response to immunotherapy (Owonikoko et al. 2019). Based on the lack of expression of neuroendocrine (NE) markers detected by immunohistochemistry (IHC), SCLC-I and/or SCLC-Y, as well as SCLC-P, are classified as non-NE subtypes, in contrast to the SCLC-A and SCLC-N phenotypes (Lo et al. 2023). These subtypes are not exclusive for each patient, and they can coexist within the same tumor. Tumor resections have shown to be positive for up to three of the lineage-specifying transcription factors, suggesting cellular plasticity across different phenotypic states (Baine et al. 2020; Qu et al. 2022; Wu et al. 2021). Ireland et al*.* demonstrated an evolution from the NE forms (SCLC-A and SCLC-N) to SCLC-Y driven by c-MYC with subsequent Notch signaling activation (Ireland et al. 2020). The Notch signaling pathway can promote the conversion of NE to non-NE states by inducing REST expression (Lim et al. 2017). YAP1 activation independently of Notch signaling can also drive REST expression, facilitating cell fate conversion (Wu et al. 2021). These transdifferentiation mechanisms promote the coexistence of diverse cellular subpopulations within a single case, reducing therapy response rates and promoting tumor progression (Schwendenwein et al. 2021; Gay et al. 2021; Gutiérrez et al. 2023).

The developmental transcription factor ONECUT2 (One cut domain family member 2; OC2) has been associated with tumor cell proliferation, epithelial-mesenchymal transition (EMT) and metastasis in several cancer types (Ma et al. 2022; Sunita Prajapati et al. 2024; Zamora et al. 2025; Rotinen et al. 2018). OC2 has been shown to be a druggable master regulator in metastatic castration-resistant prostate cancer (mCRPC) and a driver of lineage plasticity in adenocarcinoma and drug-resistant NE phenotypes (Rotinen et al. 2018; Qian et al. 2024). This transcription factor has also been postulated as a driver of cell fate in breast cancer by promoting a conversion from luminal to basal subtypes, thus emerging as a potential drug target for distinct cell states within breast tumors (Zamora et al. 2025). In this study we investigate whether OC2 plays a role in SCLC by promoting cell plasticity, heterogeneity, and tumor resistance. We demonstrate that OC2 serves as a survival factor in SCLC, and that its activity can be suppressed by a small molecule inhibitor, offering a potential strategy for targeting highly plastic SCLC tumors.

## Methods

### Cell lines culture

SCLC cells were cultured at 37 °C and 5% CO_2_ in a humidified incubator. NCI-H69, NCI-H510, NCI-H82, and DMS53 cells were grown in RPMI 1640 culture medium (Gibco) supplemented with 10% FBS and 1% penicillin/streptomycin (Gibco). The murine RP cell line (also called 5B) was obtained from Dr. Julien Sage. This is a SCLC neuroendocrine cell line characterized by loss of Rb1 and Tp53 (Schaffer et al. 2010). RP cells were grown in RPMI 1640 supplemented with 10% FetalClone III (Hyclone) and 1% penicillin/streptomycin. HEK-293T cells were maintained in DMEM (Gibco) supplemented with 10% FBS and 1% penicillin/streptomycin. Human cell lines were obtained from the American Type Culture Collection (ATCC) and tested negative for mycoplasma contamination.

### Antibodies and reagents

Anti-OC2, rabbit, polyclonal (Sigma, HPA057058, lot no. A104348) (1:100) was used for the IHC studies; anti-OC2, rabbit, polyclonal (Sigma, HPA057058, lot no. A104348) (1:1000) and anti-OC2, rabbit, polyclonal (Proteintech, 21916–1-AP, lot no. 00114937) (1:1000) were used for western blot. The following antibodies were also used for western blot: anti-ASCL1 (Abcam, AB211327, lot no. 12) (1:1000), anti-NEUROD1 (Abcam, AB109224, lot no. 1016722–2) (1:1000), anti-YAP (Cell Signaling, 14074, lot no. 5) (1:1000), anti-c-MYC (Cell Signaling, 5605, lot no.16) (1:1000), anti-β-actin, mouse, monoclonal (Sigma A5441, lot no. 0000126950) (1:2000); anti-rabbit IgG, HRP-linked (Cell Signaling 7074, lot no. 28) (1:5000); anti-mouse IgG, HRP-linked (GE-Healthcare NA931, lot no. 17271474) (1:5000). The reagents used were doxycycline hyclate (Sigma, D9891), CSRM617 (Sigma, SML2608), cisplatin (Sigma, 232120), and etoposide (Sigma, E1383).

### OC2 expression profile

Differential OC2 protein expression patterns in SCLC patients were analyzed using the SCLC U Cologne cohort (George et al. 2015) (Dataset in cBioportal (Gao et al. 2013; Cerami et al. 2012), accessed July 2, 2024) and the GSE40275 (Kastner et al. 2012), GSE30219 (Rousseaux et al. 2013), GSE149507 (Cai et al. 2021), and GSE60052 (Jiang et al. 2016) cohorts (Datasets in Gene Expression Omnibus (GEO) (Clough and Barrett 2016), accessed July 2, 2024). The U Cologne cohort (George et al. 2015) includes 81 human primary SCLC tumor specimens and clinical data of the patients. The GSE40275 cohort (Kastner et al. 2012) contains 19 SCLC and 43 normal lung samples; the GSE30219 (Rousseaux et al. 2013) contains 21 SCLC and 14 normal lung samples; the GSE149507 (Cai et al. 2021) contains 18 SCLC and 18 normal lung samples; the GSE60052 (Jiang et al. 2016) contains 75 SCLC and 7 normal lung samples. For the OC2 dependency analysis in different SCLC cell lines, ONECUT2 CRISPR Screening Data from the DepMap portal (Tsherniak et al. 2017) was used (accessed June 20, 2024).

### Lentiviral constructs

Lentivirus were generated cotransfecting the pCMV-Delta-8.2 (Addgene, 12263) packaging plasmid and the pCMV-VSVG plasmid (Addgene, 8454) into HEK-293T cells using Lipofectamine 2000 (Invitrogen). The empty vector pLenti-C-mGFP-P2A-Puro (Origene, PS100093) was used as a control and the OC2 plasmid Lenti-ORF clone of ONECUT2 (mGFP-tagged)-Human one cut homeobox 2 (Origene, RC211951L4) was used for constitutive expression of this protein. The inducible OC2 expression plasmid was obtained by cloning the full length OC2 cDNA (NM_004852) and the enhanced GFP (E-GFP) into the pCW57-MCS1-P2A-MCS2 (Neo) (Addgene, 89180). Medium was changed every 24 h and the 48 h and 72 h supernatants were filtered with a 0.45 μm PVDF filter syringe for transduction of the recipient cells. SCLC cells were transduced in the presence of 10 μg mL^−1^ of polybrene (Millipore). To obtain the constitutive and inducible lines, cells were selected with 2.5 μg mL^−1^ puromycin (Gibco) or 0.5 mg mL^−1^ geneticin G418 (Gibco), respectively. The experiments to knockdown OC2 with shRNAs were performed as previously described (Rotinen et al. 2018).

### Immunohistochemistry

Immunohistochemical staining was used to assess OC2 and synaptophysin (SYP) expression in tissue and cells grown on microscope slides, respectively. For OC2 analysis, a SCLC tissue microarray (TMA) (LC10010d, US Biomax Inc.) containing 40 lung small cell carcinomas and 10 normal lung tissues was deparaffinized and rehydrated. SYP expression in OC2 overexpressing DMS53 cells was analyzed by fixation in 3–4% formalin, followed by rinsed in 70% alcohol and incubation in 0,1% PBST (Tween-20). Endogenous peroxidase was quenched with 3% H_2_O_2_ for 10 min in both TMA and cells. Later, in tissue samples microwave antigen retrieval was carried out with EDTA buffer (EnVision™ FLEX Target Retrieval Solution, High pH, Dako Omnis) twice for 10 min. Normal goat serum at 5% in TBS-Tween (Wash buffer, Dako) was used to block non-specific binding sites for 30 min. Sections were incubated with primary antibody (anti-OC2, 1:100; HPA057058, Sigma and anti-SYP, 1:100, MA5-16402, ThermoFisher) overnight at 4 °C. Detection was performed with ENVISION HRP system (Dako). The peroxidase activity was visualized with diaminobenzidine. Finally, sections were washed, lightly counterstained with haematoxylin, dehydrated and mounted. Omission of primary antibody was used as a negative control. Staining scores were established by semiquantitative analysis as previously described (Martínez-Terroba et al. 2018). The extension of the staining was scored as the percentage of positive cells (0–100%) and the intensity of the staining was assessed using a 4-value scoring system (0 = below the level of detection, 1 = weak, 2 = moderate and 3 = strong). A final H score was calculated by adding the product of the percentage cells stained at a given staining intensity (0–100) and the staining intensity (0–3). Representative images of low, intermediate, and high OC2 expression in SCLC are shown in Supplementary Fig. 1A.

### RNA-sequencing and gene set enrichment analysis

Total RNA was extracted using the RNeasy Kit (Qiagen). Quality control analysis, library preparations and sequencing were performed at the CIMA Genomics Core (Center for Applied Medical Research, University of Navarre) and data normalization and processing at the CIMA Bioinformatics Platform as described in Zamora et al. (2025). Differentially expressed genes (DEGs) were selected with adjusted *P*-value cut off *P* < 0.001. Further functional and clustering analyses and graphical representations were performed using R/Bioconductor, MSigDB (https://CRAN.R-project.org/package=msigdbr) and clusterProfiler (Yu et al. 2012). Data are publicly available in GEO database with the accession number GSE280219.

### The lungNENomics cohort

The combined lungNENomics cohort of the Rare Cancers Genomics (RCG) team at IARC/WHO consists of a novel, currently unpublished cohort of over 400 lung NE tumor patients (Mathian et al. 2024, Sexton-Oates et al. *in prep*) plus public datasets reprocessed by the RCG team. The public datasets of the cohort were obtained from the European Genome-Phenome Archive (EGA) based on the following publications: Fernandez-Cuesta et al. (2014) (accession number: EGAS00001000650), George et al*. (*2015) (EGAS00001000925), George et al*.* (2018) (EGAS00001000708), Alcala et al*.* (2019) (EGAS00001003699), Laddha et al*.* (2019) (GSE118131), Miyanaga et al. (2020) (GSE142186), and Dayton et al*.* (2023) (EGAS00001005752). In total, this combined cohort included 497 RNA-Seq samples, among which 349 had histological type information (from central pathology review). All samples were processed or reprocessed using IARC’s open source bioinformatic pipelines (https://github.com/IARCbioinfo/) which performs quality and adapter sequence trimming, reads mapping with software STAR (Dobin et al. 2013), local realignment and base quality score recalibration, and expression quantification with software StringTie (Pertea et al. 2015). See Alcala et al. (2024) for full details about data processing and quality controls.

### Western blot analysis

Cells were lysed in RIPA buffer (Sigma, R0278) supplemented with protease inhibitors (Roche, cOmplete Mini, 11836170001). Protein concentration was measured using Coomassie Plus (Bradford) Assay Kit (Thermo, 23236). Protein lysates were separated in 4–20% SDS-PAGE (Bio-Rad Laboratories) and PVDF membranes were used for the transference. Prior to the 16 h incubation with the primary antibody, the membranes were blocked with 5% BSA. Membranes were washed with PBST (0.1% Tween-20) and incubated with an HRP-conjugated secondary antibody. After washing with PBST, the protein bands were detected by chemiluminescence (SuperSignal West Pico PLUS or West Atto Ultimate Sensitivity Chemiluminescent Substrate, ThermoFisher Scientific).

### Proteomic analysis

Cell pellets were homogenized in a lysis buffer (7 M urea, 2 M thiourea, 50 mM dithiothreitol (DTT)), supplemented with protease inhibitors (Roche, cOmplete Mini, 11836153001) and phosphatase inhibitors (Roche, PhosSTOP, 4906845001). Lysates were centrifuged at 20,000 g (1 h, 15 °C), and the resulting supernatant was quantified with the Bradford assay kit (BioRad). Proteins (600 µg) were reduced with DTT (final concentration of 20 mM; room temperature, 30 min), alkylated with iodoacetamide (final concentration of 30 mM; room temperature, 30 min in the dark), diluted to 0.9 M with ABC and digested with trypsin (Promega; 1:20 w/w enzyme protein ratio, 18 h, 37 °C). Protein digestion was interrupted by acidification (pH < 6, acetic acid), and the resulting peptides were cleaned-up using Pierce™ Peptide Desalting Spin Columns (ThermoFisher). A 10 µg aliquot of cleaned-up peptides from protein digestion was set aside for total protein analyses.

For data independent acquisition (DIA)-mass spectrometry, dried down peptide samples were reconstituted with 2% acetonitrile—0.1% formic acid, spiked with internal retention time peptide standards (iRT, Biognosys), and quantified by NanoDropTM spectrophometer (ThermoFisher Sci.) prior to LC–MS/MS analysis using an EVOSEP ONE system coupled to an EZ-Exploris 480 mass spectrometer (Thermo Fisher Sci.). Peptides were resolved using C18 Performance column (75 µm × 15 cm, 1.9 µm particles; Evosep) with a predefined Xcalibur Whisper100 20 SPD (58 min, IonOpticks Aurora Elite, EV1112) method. Peptides were ionized using 1.6 kV spray voltage at a capillary temperature of 275 °C. Sample data were acquired in DIA mode with full MS scans (scan range: 400 to 900 m/z; resolution: 60,000; maximum injection time: 22 ms; normalized AGC target: 300%) and 24 periodical MS/MS segments applying 20 Th isolation windows (0.5 Th overlap: Resolution: 15,000; maximum injection time: 22 ms; normalized AGC target: 100%). Peptides were fragmented using a normalized HCD collision energy of 30%.

Mass spectrometry data files were analyzed using Spectronaut (Biognosys) by direct DIA analysis (dDIA). MS/MS spectra were searched against the Uniprot proteome reference from human database using standard settings. Enzyme was set to trypsin in a specific mode. Carbamidomethyl (C) was set as a fixed modification, and oxidation (M), acetyl (protein N-term), deamidation (N), and Gln- > pyro-Glu as variable modifications for total protein analysis. Identifications were filtered by a 1% Q-value. The obtained quantitative data were exported to Perseus software (version 1.6.15.0) (Tyanova et al. 2016) for statistical analysis and data visualization. For total protein analysis, unpaired Student’s t-test was used for direct comparisons. Statistical significance was set at *P*-value lower than 0.05 in all cases.

Differential proteins were assessed using Metascape (Zhou et al. 2019) cell component term using default settings (min. overlap: 3, min. enrichment:|1.5|, *P* < 0.05), and false discovery rate (FDR) adjusted *P* < 0.05. Protein interactomes and activation ranking were analyzed using QIAGEN’s Ingenuity Pathway Analysis (IPA; QIAGEN Redwood City) (Krämer et al. 2014). Mass-spectrometry data and search results files were deposited in the Proteome Xchange Consortium via the JPOST partner repository (Moriya et al. 2019) with the identifier PXD057006 for ProteomeXchange and JPST003432 for jPOST.

### Quantitative PCR

Total RNA was extracted (RNeasy Kit, Qiagen) and subjected to One-Step Real Time RT-PCR in a CFX-Connect Real Time PCR Detection System (Bio-Rad) with AgPath-ID One-Step RT-PCR Reagents (ThermoFisher) and TaqMan probes for: ONECUT2 (Hs00191477_m1), ASCL1 (Hs00269932_m1), NEUROD1 (Hs00159598_m1), YAP1 (Hs00902712_g1), REST (Hs00958502_m1), c-MYC (Hs00153408_m1), l-MYC (Hs00420495_m1). Data were analyzed using the 2^−ΔΔCT^ method. ACTB and TUBB TaqMan probes (Hs01060665_g1 and Hs00742828_s1, respectively) were used for normalization.

### Proliferation assays

Cell viability was measured with CellTiter-Glo Luminescent Cell Viability Assay (Promega). Briefly, 10,000 cells per well were plated in 96-well plates. T0 readings were collected after 24 h. To induce OC2 expression cells were treated with 50 ng/mL doxycycline. Measurements were performed every 24 h during 6 consecutive days. Non-induced cells were used as control.

### Dose–response curves and IC50

1,000 cells per well were plated in a 96-well plate. T0 readings were collected after 24 h, and cells were exposed to vehicle (1% DMSO) or compound CSRM617 at twofold concentration ranging from 100 μM to 0.2 μM. CellTiter-Glo Luminescent Cell Viability Assay was used to measure viability 48 h post-treatment. Dose–response curves and IC50 values were generated using GraphPad Prism version 9 software. SynergyFinder 3.0 (Ianevski et al. 2022) was used to predict the synergistic effect of CSRM617 and cisplatin. Cells were treated with the OC2 inhibitor CSRM617 at a final concentration of 100, 50, 25, 12.5 or 6.25 µM and with the combination of cisplatin and etoposide (1:2) at 25, 12.5, 6.25, 3.12 or 1.56 µM for cisplatin and 50, 25, 12.5, 6.25 or 3.12 µM for etoposide for 48 h.

### Apoptosis quantification

Cells were stained with APC-Annexin V and 7-Aminoactinomycin D (7-AAD) (BioLegend). Apoptotic cell detection was performed using FACSCanto II Flow Cytometer (BD Biosciences) and software Flowjo v10 (https://www.flowjo.com) was used for data analysis. Cells were gated by FSC-A x SSC-A to exclude debris and then by FSC-H x FSC-W following SSC-H x SSC-W to exclude cell doublets.

### Statistics and reproducibility

The normality of the data was assessed using a Shapiro–Wilk test. Data with normal distribution followed a two-tailed Student's t-test. Non-normal samples were analyzed using a Wilcoxon two-tailed rank-sum test. For every comparison *P* < 0.05 was considered statistically significant and, *P* < 0.01 highly significant. Excel and the R package (RSudio 2023.06.0 + 421) were used for all statistical tests. Data was visualized with GraphPad (version 9) and BioRender (www.biorender.com) software.

## Results

### OC2 expression is associated with lymph node metastasis and clinical stage in SCLC patients

**Fig.1:**
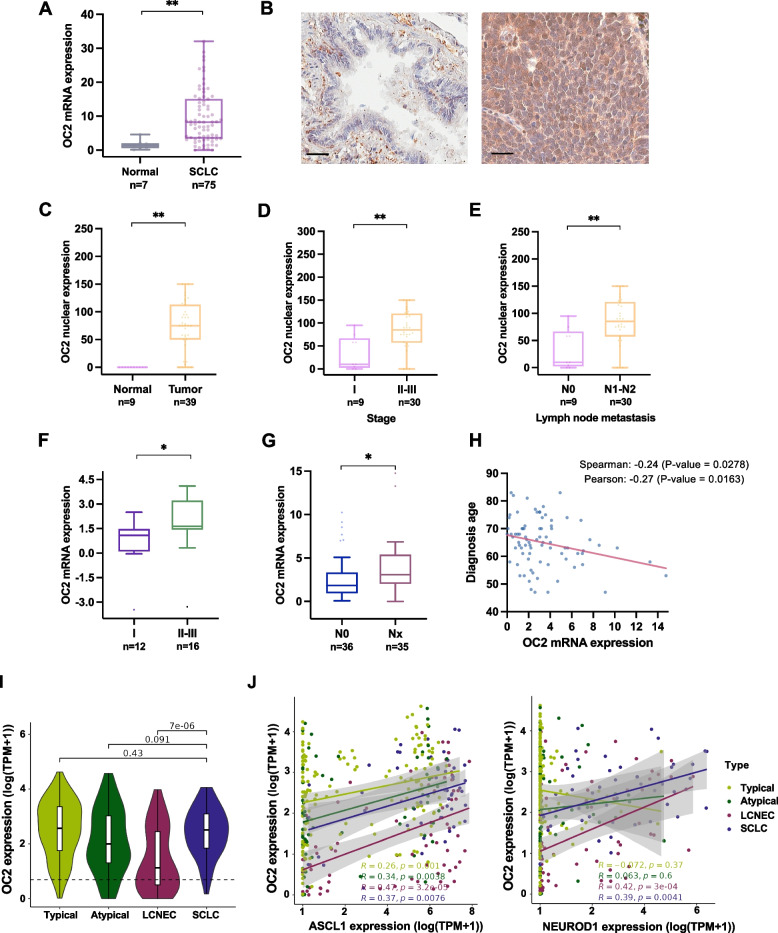
Association between OC2 expression and clinicopathological features in patients with SCLC.** A** OC2 mRNA expression (TPM normalized) in normal lung tissue compared to SCLC from the GSE60052 cohort (Jiang et al. 2016). The boxes show the 25–75^th^ percentile range, and the center line is the median. Whiskers extend from the minimum and maximum values. *P*-values were obtained from Wilcoxon two-tailed rank-sum test. **B**,** C** Representative IHC images (**B**) and quantification (**C**) of OC2 expression in benign (left) and SCLC tissue (right). OC2, brown. Scale bar, 50 µm. **D**,** E** Association of stage (**D**) and lymph node metastasis (**E**) with OC2. For (**C-E**) boxplots of intensity levels of nuclear OC2 expression assessed by IHC using a SCLC TMA are shown. The boxes show the 25–75^th^ percentile range, and the center line is the median. Whiskers extend from the minimum and maximum values. *P*-values were obtained from Wilcoxon two-tailed rank-sum test.** F** Association of stage with OC2 mRNA expression (arbitrary units) from SCLC tumors from the Lung Cancer Transcriptome Atlas (LCTA, *in prep*) cohort. **G** Association of lymph node metastasis with OC2 mRNA expression (FPKM, Fragments Per Kilobase per Million mapped fragments) from SCLC tumors from the U Cologne cohort (George et al. 2015). Nx = N1, N2 and N3 grouped. For (**F, G**) the boxes show the 25–75^th^ percentile range, and the center line is the median. Whiskers show 1.5 times the interquartile range (IQR) from the 25^th^ or 75^th^ percentile values. For (**F**), *P*-values were obtained from unpaired two-tailed Student´s t-test. For (**G**), Wilcoxon two-tailed rank-sum test was performed.** H** Correlation between OC2 mRNA expression (FPKM) and age of patients at diagnosis in the U Cologne cohort (George et al. 2015). **I** OC2 log2 (tpm + 1) mRNA expression in different lung NE neoplasms from the combined lungNENomics cohort. *P*-values were obtained from Wilcoxon two-tailed rank-sum test. **J** Pearson correlation between OC2 and ASCL1 (left) and NEUROD1 (right) mRNA expression in the lungNENomics cohort. For every comparison, *P* < 0.05 was considered significant. *P < 0.05, **P < 0.01

OC2 expression is elevated in cancers such as prostate, colorectal, ovarian, hepatocellular, and non-small cell lung cancer. High OC2 levels are associated with tumor cell proliferation and metastasis (Sunita Prajapati et al. 2024). In SCLC, OC2 mRNA expression is also significantly higher in tumor tissue compared to adjacent normal lung tissue, as observed in the GSE60052 (Jiang et al. 2016), GSE40275 (Kastner et al. 2012), GSE30219 (Rousseaux et al. 2013), and GSE149507 (Cai et al. 2021) cohorts (Fig. 1A and Supplementary Fig. 1B). Consistently, OC2 immunostaining of a SCLC TMA that includes non-tumor lung tissue and malignant small cell lung carcinoma, revealed that OC2 is highly expressed in the nucleus and cytoplasm of tumor cells, while non-tumor tissues showed low or no expression of OC2 (Fig. 1B, C and Supplementary Fig. 1C). The relationship between OC2 expression and clinicopathological parameters, including age and TNM cancer stage, was next examined by quantitative analysis of OC2 immunostaining intensity. The results showed elevated nuclear and cytoplasmic OC2 expression in patients with late-stage tumors and lymph node metastasis compared to early-stage tumors (Fig. 1D, E and Supplementary Fig. 1D). Heightened OC2 mRNA levels are also associated with advanced tumor stage and nodal metastasis in the Lung Cancer Transcriptome Atlas (LCTA, *in prep*) and the U Cologne (George et al. 2015) cohorts (Fig. 1F, G). In the U Cologne cohort, OC2 mRNA expression is negatively correlated with patient age at diagnosis (Fig. 1H).

We previously showed that among all cell lines included in the Cancer Cell Line Encyclopedia (CCLE) database, OC2 mRNA levels are highest in SCLC (Rotinen et al. 2018). In accordance with this, in the combined lungNENomics study cohort including 349 transcriptomes of lung NE neoplasms from published cohorts and a novel cohort (George et al. 2015; Fernandez-Cuesta et al. 2014; George et al. 2018; Alcala et al. 2019; Laddha et al. 2019; Miyanaga et al. 2020; Dayton et al. 2023), OC2 expression is relatively high in lung NE neoplasms including SCLC (> 1 Transcripts Per Million (TPM) in 88% of tumors among which 50/51 SCLC; Fig. 1I). A significant positive correlation of OC2 and ASCL1 or NEUROD1 mRNA levels in SCLC is observed in the lungNENomics cohort (Fig. 1J).

### OC2 promotes NE to non-NE transition of SCLC cells

**Fig. 2:**
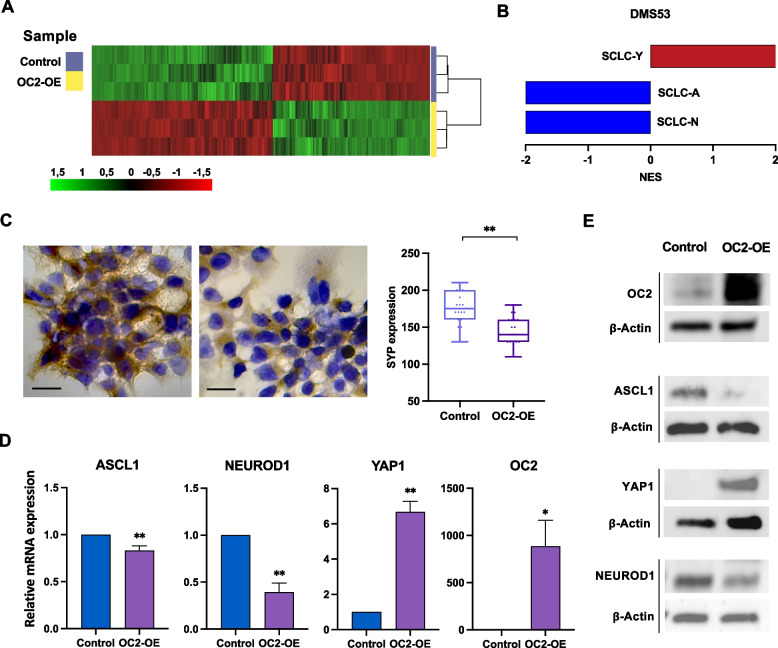
OC2 modulates plasticity from NE to non-NE phenotypes in the DMS53 cell line.** A** Heatmap showing DEGs (adjusted *P*-value < 0.001) after OC2 enforced expression in DMS53 cells analyzed by hierarchical clustering. Three independent RNA-Seq experiments were performed per condition. **B** Plot showing upregulated (red bars) and downregulated (blue bars) SCLC subtype-specific gene signatures (Ireland et al. 2020) after OC2 enforced expression. **C** Immunohistochemical staining of SYP in OC2 overexpressing DMS53 cells. The boxes show the 25–75^th^ percentile range, and the center line is the median. Whiskers extend from the minimum and maximum values. *P*-values were obtained from Wilcoxon two-tailed rank-sum test. **D**,** E** mRNA (**D**) and protein levels (**E**) of OC2, ASCL1, NEUROD1 and YAP1 after OC2 enforced expression in DMS53 cells. For (**D**) qRT-PCR results were normalized using β-actin. The mean + SEM from three independent experiments is shown. Unpaired two-tailed Student’s t-test, *P < 0.05, **P < 0.01. For (**E**) representative blots from three independent experiments are shown. **F** Plot showing upregulated (red bars) non-NE gene signatures and downregulated (blue bars) NE gene signatures (Cai et al. 2021; Zhang et al. 2018)

To study the role of OC2 in SCLC, we enforced OC2 expression in the widely studied NE-SCLC DMS53 cell line (OC2 endogenous levels in this and other SCLC cell lines are shown in Supplementary Fig. 2) and performed gene expression profiling. The RNA-Seq analysis showed 9,241 DEGs (adjusted *P*-value < 0.001) (Fig. 2A). Gene set enrichment analysis (GSEA) of SCLC molecular subtypes signatures revealed a significant repression of SCLC-A (Ireland et al. 2020) and SCLC-N (Ireland et al. 2020) signatures and an enrichment of a SCLC-Y signature (Ireland et al. 2020) (Fig. 2B). Forced OC2 expression repressed mRNA levels of multiple genes that define the ASCL1 and NEUROD1 subtypes (Schwendenwein et al. 2021) (Supplementary Table 1 and Supplementary Table 2). Additionally, IHC analysis of cells with forced OC2 expression showed a significant repression of the NE marker SYP (Fig. 2C). In concordance with these findings, we observed downregulation of ASCL1 and NEUROD1 and upregulation of YAP1, both at the mRNA (Fig. 2D) and protein level (Fig. 2E). When analyzing different NE and non-NE gene signatures (Cai et al. 2021; Zhang et al. 2018) we also found a negative enrichment of NE genes and a positive enrichment of non-NE genes (Fig. 2F). Taken together these data suggest that OC2 might be involved in the conversion from NE subtypes to the non-NE molecular subtype YAP1.

**Fig. 3:**
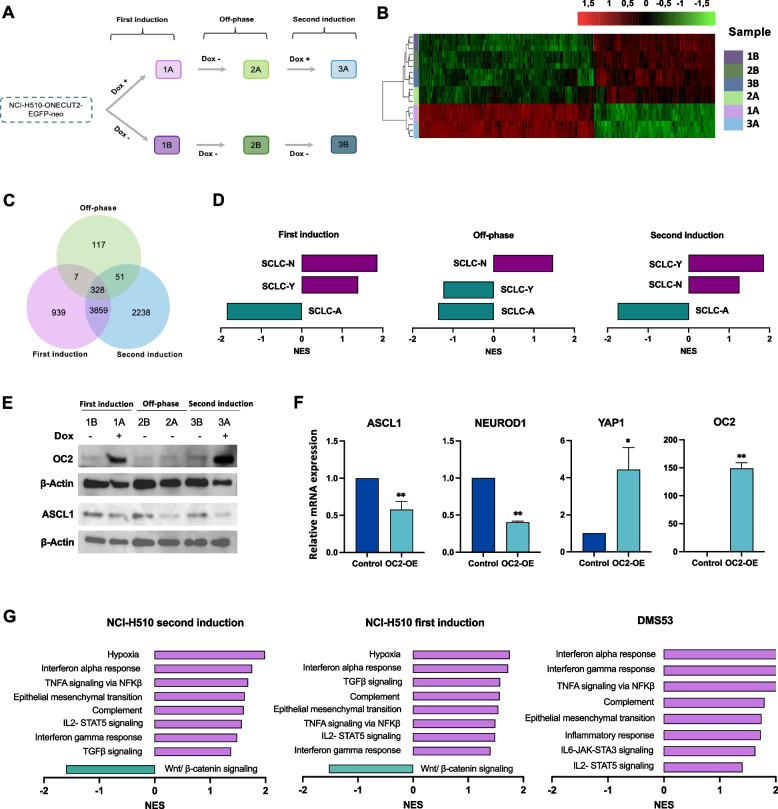
Inducible expression of OC2 in NCI-H510 cells leads to a phenotypic transdifferentiation towards a non-NE subtype.** A** Scheme of the experimental protocol of induction/off-phase/induction of OC2 expression in the NCI-H510 cell line. Cells were cultured with 50 ng/mL doxycycline (dox) for 5 days (first induction). Subsequently, the medium was replaced with complete RPMI for 6 days (off-phase). Finally, doxycycline was added again for 5 days (second induction).** B** Heatmap showing DEGs (adjusted *P*-value < 0.001) during the first induction (samples 1A and 1B), off-phase (samples 2A and 2B), and second induction (samples 3A and 3B) analyzed by hierarchical clustering. Three independent RNA-Seq experiments were performed per condition. **C)** Venn diagram of the overlapping DEGs during the induction/off-phase/induction experiment.** D** Plots showing upregulated (purple bars) and downregulated (green bars) SCLC subtype-specific gene signatures (Ireland et al. 2020) in NCI-H510 cells. **E** Immunoblot showing OC2 and ASCL1 expression during the induction/off-phase/induction experiment. **F** YAP1, ASCL1 and NEUROD1 mRNA expression after constitutive OC2 overexpression in NCI-H510 cells. Results were normalized using β-actin. The mean + SEM from at least three independent experiments is shown.** G** MSigDB Hallmark Gene Sets enriched in OC2 upregulated (purple bars) and downregulated (green bars) genes in OC2 overexpressing NCI-H510 and DMS53 cells

The observation that OC2 represses ASCL1 in DMS53, a NE-SCLC cell line suggests that OC2 may be a driver of transdifferentiation of the SCLC-A subtype. To explore this further, an induction/off-phase/induction sequence of OC2 expression was performed in the ASCL1-driven NCI-H510 cell line using a doxycycline inducible OC2-EGFP system (Fig. 3A). The RNA-Seq analysis showed a total of 6,276 DEGs (adjusted *P*-value < 0.001) after the second induction of OC2 expression. Among these genes, 3,987 were altered during the first induction and 328 remained perturbed during the off-phase (Fig. 3B, C). GSEA analysis of a SCLC-A gene signature (Ireland et al. 2020) showed a significant repression in each OC2 induction (Fig. 3D). Sustained downregulation of ASCL1 protein levels after the first and second induction of OC2 and loss of ASCL1 during the off-phase of the experiment was confirmed by western blot (Fig. 3E). Conversely, a positive enrichment of the SCLC-Y signature (Ireland et al. 2020) was observed after both inductions of OC2 (Fig. 3D). Of note, induction of the SCLC-Y signature was suppressed during the off-phase (Fig. 3D). YAP1 upregulation, together with ASCL1 and NEUROD1 downregulation, were confirmed after OC2 enforced expression in the constitutive system at the mRNA level (Fig. 3F).

The YAP1 subtype has been related to an inflamed and mesenchymal phenotype (Qu et al. 2022; Gay et al. 2021). Consistent with this notion, GSEA Hallmark pathway enrichment analysis revealed changes in EMT, inflammation, and immune related pathways (e.g. interferon (IFN) alpha and gamma responses, and TNFa/NFKB and IL-STAT) after OC2 overexpression in both NCI-H510 and DMS53 cells (Fig. 3G). These data suggest that OC2 expression can trigger a phenotypic evolution from the SCLC-A subtype towards an inflamed, SCLC-I subtype in these two SCLC cell line models. The RNA-Seq dataset also showed that the expression of POU2 F3 and several marker genes within the SCLC-P subtype (Schwendenwein et al. 2021), including AVIL and SOX9, were upregulated after the first and second OC2 induction in NCI-H510 cells (Supplementary Table 3). Surprisingly, a positive enrichment of the SCLC-N signature (Ireland et al. 2020) was also observed (Fig. 3D) and this signature remained highly expressed during the off-phase of OC2 expression. This result in the NCI-H510 cell line differs with the repression of this signature observed after constitutively enforced OC2 expression in the DMS53 cell line (Fig. 2B). These data suggest that OC2 induction promotes a shift in SCLC from ASCL1^+^ to YAP1^+^/POU2 F3^+^ stages through the NEUROD1^+^ intermediate state.

### OC2 activates c-MYC and Notch signaling in SCLC cells

**Fig. 4:**
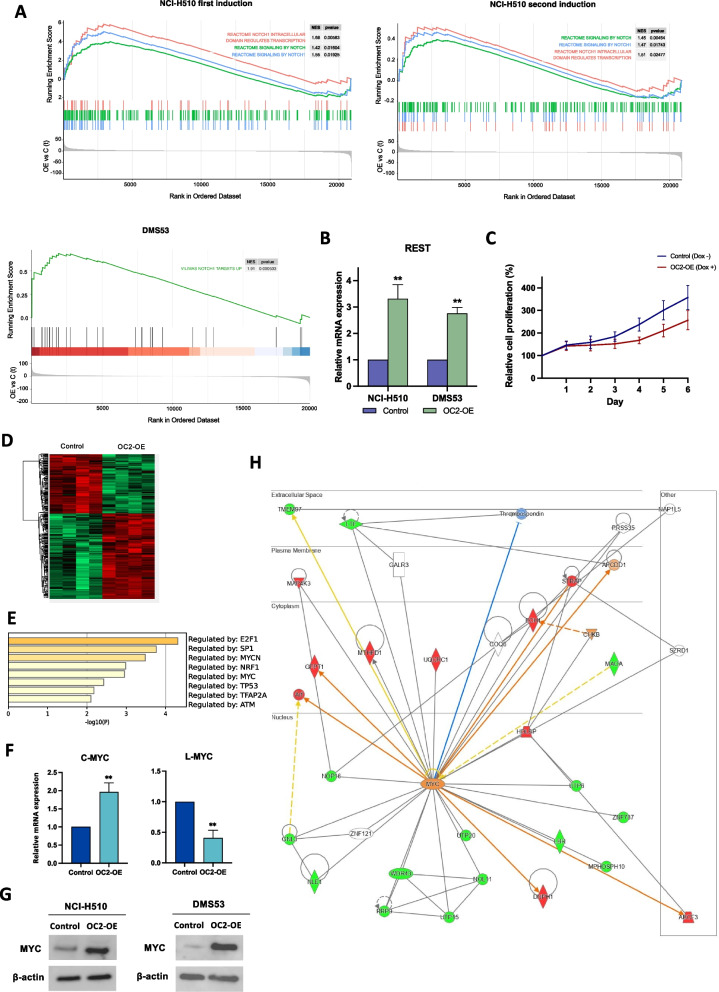
OC2 induction alters NE transdifferentiation drivers.** A** GSEA plots showing enrichment of Notch signaling signatures (Vilimas et al. 2007; Milacic et al. 2024) in NCI-H510 and DMS53 cells after OC2 inducible and constitutive overexpression, respectively. Three independent RNA-Seq experiments were performed. **B** REST mRNA expression after OC2 enforced expression in the DMS53 and NCI-H510 cell lines. The mean + SEM from three independent experiments is shown. Unpaired two-tailed Student’s t-test, **P < 0.01. Results were normalized using β-actin. **C** Cell proliferation curve showing OC2 induced *vs.* control NCI-H510 cells. Proliferation was calculated relative to T0 number of cells. The values shown are the mean ± SEM from five independent experiments. **D** Heatmap showing DEPs (*P*-value < 0.05) after OC2 induction in NCI-H510 cells. **E** Heatmap of transcription factors generated with Metascape employing the TRRUST method using DEPs between control and OC2 overexpressing NCI-H510 cells. **F** c-MYC and l-MYC mRNA expression after OC2 constitutive overexpression in the NCI-H510 cell line. The mean + SEM from four independent experiments is shown. Unpaired two-tailed Student’s t-test, **P < 0.01. Results were normalized using β-actin. **G** c-MYC protein expression levels detected by western blot after OC2 overexpression in the NCI-H510 and DMS53 cell lines. β-actin was used as control. **H** Protein networks and their cellular localization obtained in response to OC2 induction in the NCI-H510 cell line. Networks are arranged in the form of nodes (proteins) and lines (biological relationships between nodes). The proteins in green are overexpressed and those in red are repressed. The proteins in white do not belong to our dataset but are included in the database. In orange, the program predicts protein activity activation, and in blue, inhibition. The solid lines connecting the nodes represent direct interactions, while the dashed lines represent indirect interactions. The color of the lines indicates activation if orange, inhibition if blue, inconsistent findings if yellow, and unpredictable effects if gray. The shape of the nodes denotes the protein function: enzymes (diamond), transcriptional regulators (oval), kinases (inverted triangle), peptidases (horizontally oriented diamond), nuclear receptors (rectangle), transporters (trapezoid), others (circle). Image obtained from Ingenuity

It has been shown that evolution of SCLC into non-NE states can be driven by c-MYC induction of the Notch/Rest signaling pathway (Ireland et al. 2020). Consistent with this concept, GSEA analysis after OC2 enforced expression showed significant activation of Notch signaling in both DMS53 and NCI-H510 cells (Fig. 4A). The activation of the Notch pathway is reversible since it ceased during the off-phase in the inducible system. REST upregulation was detected in both backgrounds (Fig. 4B). Aligned with the notion that Notch activation slows the growth of SCLC cells (Qu et al. 2022), our results show that OC2 overexpression in NCI-H510 cells reduces growth pattern compared to control cells (Fig. 4C). In addition, we also observed repression of the Wnt/B-catenin pathway in the NCI-H510 modified cell line (Fig. 3G).

In order to better understand the role of OC2 in promoting non-NE fates, we next performed a proteomic analysis after OC2 enforced expression in the NCI-H510 cell line. As shown in Fig. 4D and Supplementary Fig. 3, OC2 induction resulted in 838 Differentially Expressed Proteins (DEPs) (*P*-value < 0.05). As expected, ASCL1 protein expression and several SCLC-A subtype markers (Schwendenwein et al. 2021) were found to be downregulated (Supplementary Table 4). Among the DEPs, Metascape TRRUST analysis identified enrichment of c-MYC targets (*P*-value = 0.001) (Fig. 4E). c-MYC upregulation after OC2 overexpression was validated by RT-qPCR (Fig. 4F) and western blot (Fig. 4G). Expression of its paralog, l-MYC, which is restricted to the SCLC-A subtype, was found downregulated at the mRNA level (Fig. 4F). Ingenuity Pathway Analysis (IPA) of DEPs also predicted c-MYC activation, suggesting a role of c-MYC as a central node of the OC2 modulated network (Fig. 4H).

### OC2 depletion decreases cell viability and induces apoptosis of NE-SCLC cell lines

**Fig. 5:**
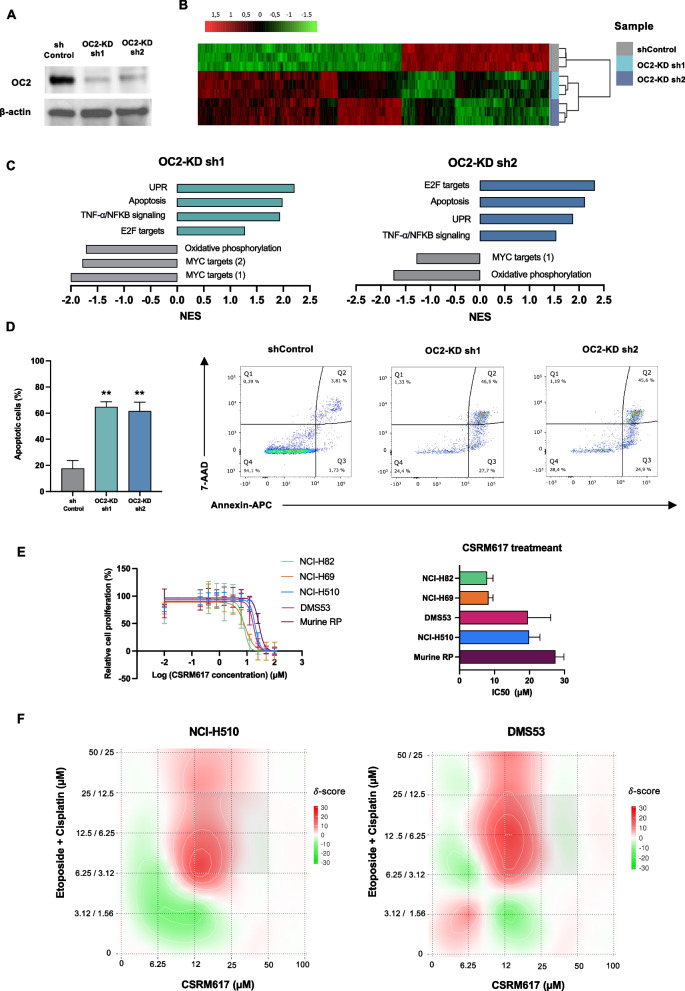
OC2 depletion decreases cell viability and induces apoptosis of a subset of SCLC cell lines.** A** OC2 protein expression levels detected by western blot after OC2 silencing with shRNAs. β-actin was used as control. **B** Heatmap showing DEGs (adjusted *P*-value < 0.001) after OC2 depletion in NCI-H510 cells. **C** MSigDB Hallmark Gene Sets upregulated (blue bars) and downregulated (grey bars) in OC2 depleted NCI-H510 cells. **D** Cell death caused by OC2 silencing measured by flow cytometry. The graph shows the mean + SEM from three independent experiments. Unpaired two-tailed Student´s t-test, * = *P* < 0.05, ** = *P* < 0.01. **E** Dose–response curves (left) and IC50 values (right) for compound CSRM617 in mouse and human SCLC cell lines after 48 h treatment with CSRM617. The values shown are the mean ± SEM from three independent experiments. **F** SynergyFinder software was used to calculate the synergy score according to the HSA model. Positive (red) or negative (green) synergy scores indicate synergistic and antagonistic effects, respectively. The shown values are the mean from three independent experiments

OC2 has been postulated as a survival factor and its inhibition has been shown to reduce tumor burden and progression in other tumor types such as breast (Zamora et al. 2025) and prostate (Rotinen et al. 2018). CRISPR screening data from DepMap identifies NCI-H510 among the SCLC cell lines with a higher OC2 dependency (Supplementary Fig. 4A). To assess the function of OC2 in SCLC viability, OC2 levels were depleted in the NCI-H510 cell line with two independent shRNAs (Fig. 5A). Gene expression profiling performed on these cells identified 1,466 common DEGs for both hairpins (adjusted *P*-value < 0.001) (Fig. 5B and Supplementary Fig. 4B). As expected, a significant repression of hallmark MYC target genes was observed (Fig. 5C). OC2 depletion also resulted in a negative enrichment of oxidative phosphorylation pathways. In contrast, we obtained positive enrichments of the Unfolded Protein Response (UPR), the inflammatory stress (TNF-a/NFKB) and the apoptosis pathways (Fig. 5C). Extensive cell death in the NCI-H510 cultures after OC2 depletion was also observed by flow cytometry (Fig. 5D). Treatment with CSRM617, a small molecule inhibitor of OC2 (Rotinen et al. 2018), reduced viability of several human SCLC-A cell lines (Fig. 5E). Moreover, the combination of CSRM617 and EP (etoposide + cisplatin) exhibited a synergistic effect in NCI-H510 and DMS53 cells (Fig. 5F and Supplementary Fig. 4C). Treatment with the OC2 inhibitor also reduced viability of the NEUROD1-driven NCI-H82 cell line, as well as the murine RP cell line, which is characterized by the loss of Rb1 and Tp53 and derived from a GEMM model (Schaffer et al. 2010) (Fig. 5E). Taken together these data suggest that OC2 might be a therapeutic target in the NE molecular subtypes of SCLC.

## Discussion

The identification of molecular subtypes and underlying mechanisms for their generation, is critical to predict therapeutic vulnerabilities and drug resistance in SCLC. At present, the transdifferentiation mechanisms governing the dynamic evolution among the different SCLC subtypes remain largely unknown. In this study, we demonstrate that the transcription factor OC2 is a driver of phenotypic transdifferentiation from the classic NE-SCLC subtypes to the more chemoresistant non-NE subtypes. Our data are consistent with a notion that expression of OC2 can lead to phenotypic diversity and the co-existance of NE and non-NE subtypes of SCLC in the same tumor. Since the non-NE SCLC subtypes possess heightened chemoresistance, the expression of OC2 may be one of the factors responsible for the rapid development of treatment resistance in SCLC. Additionally, we experimentally demonstrate that OC2 acts as a survival factor in SCLC cell lines and its inhibition with a small molecule inhibitory drug might be a potential therapeutic strategy in this highly lethal cancer type.

The role of OC2 in cell plasticity has been previously described in breast and prostate cancer (Zamora et al. 2025; Rotinen et al. 2018; Guo et al. 2019). In mCRPC, a lethal variant of NE prostate cancer, OC2 drives the transition from androgen receptor (AR)-dependent, hormone sensitive prostate cancer to AR-indifferent mCRPC by activating a neural differentiation program (Rotinen et al. 2018). In contrast to prostate cancer, where lineage plasticity is manifested by transdifferention from adeno- to NE carcinoma, in SCLC OC2 directs a reverse transition from NE to non-NE phenotypes (Rubin et al. 2020). Specifically, ectopic expression of OC2 in SCLC elicits the repression of NE programs and SCLC-A gene signatures, along with the positive enrichment of non-NE and SCLC-Y signatures. The dynamic evolution in SCLC from ASCL1^+^ to YAP1^+^, through a NEUROD1^+^ state, directed by c-MYC via Notch, was first described by Ireland et al*.* (Ireland et al. 2020). Our RNA-Seq results show that OC2 overexpression in NE-SCLC cell lines leads to activation of Notch signaling and activates pathways related with non-NE states. Additionally, our proteomic analysis confirms c-MYC activation upon OC2 induction in SCLC cells at the protein level. While YAP1 was initially proposed as a defining transcription factor of a distinct SCLC molecular subtype, recent studies have reclassified the YAP1-expressing subtype as non-SCLC (Ng et al. 2024), and evidence suggests that YAP1 is not a subtype-specific transcription factor in SCLC tumors (Baine et al. 2020). Nonetheless, YAP1 expression persists in several patient-derived models from circulating SCLC tumor cells (Pearsall et al. 2020), adding to the ongoing controversy. Importantly, recent work has proposed the existence of a broader inflamed and mesenchymal SCLC subtype that is not necessarily dependent on YAP1 expression, highlighting the complexity of SCLC subtype classification (Gay et al. 2021). Interestingly, our transcriptomic data show positive enrichment upon forced OC2 expression of, not only SCLC-Y signatures, but also EMT and inflammation/immune related pathways in two separate cell lines. Although the activation of these pathways is consistent in both cell lines, we observed the positive enrichment of the SCLC-N signature only in the NCI-H510 cell line upon OC2-inducible expression. This result suggests that the dynamic evolution towards a non-NE fate may depend on OC2 expression levels, and that this switch does not occur synchronously across all cells: while some cells may have reached the end of the hierarchical evolution of SCLC, others remain in intermediate stages. This concept is further supported by the reversible enrichment of the SCLC-Y signature, but not of the SCLC-A or SCLC-N signatures in our inducible NCI-H510 model system. Also supporting a progressive plastic process, more DEGs are observed after the second OC2 induction compared to the first one in this model. Together, these results reveal the common occurrence of a partial reversion of OC2-regulated gene expression. When translated to patient tumors, the multifactorial nature and dynamics of OC2-regulated gene expression may account for the high level of plasticity observed in SCLC tumors (Redin et al. 2024).

It has been suggested that the high prevalence of co-expression of lineage-specifying transcription factors could be a manifestation of intra-tumoral heterogeneity in SCLC. Co-expression of POU2 F3^+^/YAP1^+^ within a tumor has been described as the second most common combination following ASCL1^+^/NEUROD1^+^ (Qu et al. 2022). Upon overexpression of OC2 in NCI-H510 cells, we observe the enrichment of SCLC-Y signature and the upregulation of POU2 F3 together with several other markers of the SCLC-P subtype. This finding suggests that OC2 may play a broader role in the induction of non-NE lineages, driving cell fates towards SCLC-Y, SCLC-P or combined SCLC subtypes. An important next question that we are not able to resolve with the analysis of our bulk cell line data is whether the SCLC subtypes co-exist in the same cell, or whether they represent separate cell populations within the NCI-H510 cell line. The underlying reasons for the evolution of highly aggressive NE tumors towards a non-NE phenotype remain a subject of extensive debate. Although non-NE cells exhibit slower growth rates, it has been shown that these cells are more chemoresistant, and provide trophic support to the NE metastatic niche (Lim et al. 2017). Indeed, intra-tumoral heterogeneity increases upon targeted therapy acting as a mechanism of adaptive resistance, where multiple coexisting populations are found in relapsing SCLC patients (Stewart et al. 2020).

OC2 has been previously shown to act as a survival factor that can be targeted with a small molecule inhibitor in breast and prostate tumors. In line with this, our analysis of RNA-Seq data performed after OC2 silencing showed a positive enrichment in the UPR and in oxidative and inflammatory stresses, ultimately leading to the activation of apoptotic cell death. In this study we show that pharmacological inhibition of OC2 with CSRM617, a OC2 inhibitor developed by medicinal chemists in our group (Rotinen et al. 2018) effectively suppresses the growth of human and mouse cell lines representative of the ASCL1 and NEUROD1 SCLC subtypes. Several concerns have been raised regarding OC2 inhibition with this drug-like molecule, including issues related to its selectivity, the high patient-to-patient variability in OC2 expression that may impact patient response, and the potential effects of its combination with other treatments (Joglekar et al. 2020). In this study, we present new data (Supplementary Fig. 5A, B) demonstrating that OC2 silencing leads to resistance to CSRM617, while re-expression of OC2 restores sensitivity to the compound. Additionally, our data show that approximately 90% of SCLC tumors (George et al. 2015; George et al. 2024) express OC2 (Supplementary Fig. 5C) suggesting that a significant proportion of SCLC patients could potentially benefit from OC2 inhibition therapy. Importantly, we further show that compound CSRM617 acts synergistically with EP, a combination used in standard first-line therapy for SCLC. While EP is effective initially, many patients with SCLC experience a recurrence of the cancer. Over time, the cancer cells develop resistance to EP, making subsequent treatments less effective. Our data suggests that OC2 may be involved in the development of drug resistance in the NE molecular subtypes of SCLC and that OC2 suppression may serve to inhibit the emergence of treatment-resistant, non-NE subtypes of SCLC. In this context, measurement of OC2 expression can be developed as a companion diagnostic assay for predicting the response to OC2 inhibitors.

In summary, in this study we show that OC2 is associated with lymph node metastasis and heightened clinical stage in SCLC. Overexpression of this transcription factor drives SCLC plasticity from NE to non-NE phenotypes by activating c-MYC and Notch signaling. OC2 is required for cell survival in SCLC and its inhibition can suppress cell growth in NE-SCLC.

## Supplementary Information

Supplementary Material 1.

## Data Availability

Data generated or analyzed during this study are included in the manuscript (and its supplementary information files). RNA-Seq data are available in GEO database with the accession number GSE280219. Mass-spectrometry data and search results files have been deposited in the Proteome Xchange Consortium via the JPOST partner repository with the identifier PXD057006 for ProteomeXchange and JPST003432 for jPOST.
