# Supplementary material for: ONECUT2 reprograms neuroendocrine fate and is an actionable therapeutic target in small cell lung cancer
Source: Mol Med. 2025 Jun 11;31:232. doi: 10.1186/s10020-025-01267-6 (PMC12153148; doi:10.1186/s10020-025-01267-6)
Supplement: Supplementary file 1 — Supplementary Material 1. [file 10020_2025_1267_MOESM1_ESM.pdf]

Supplementary Figure 1

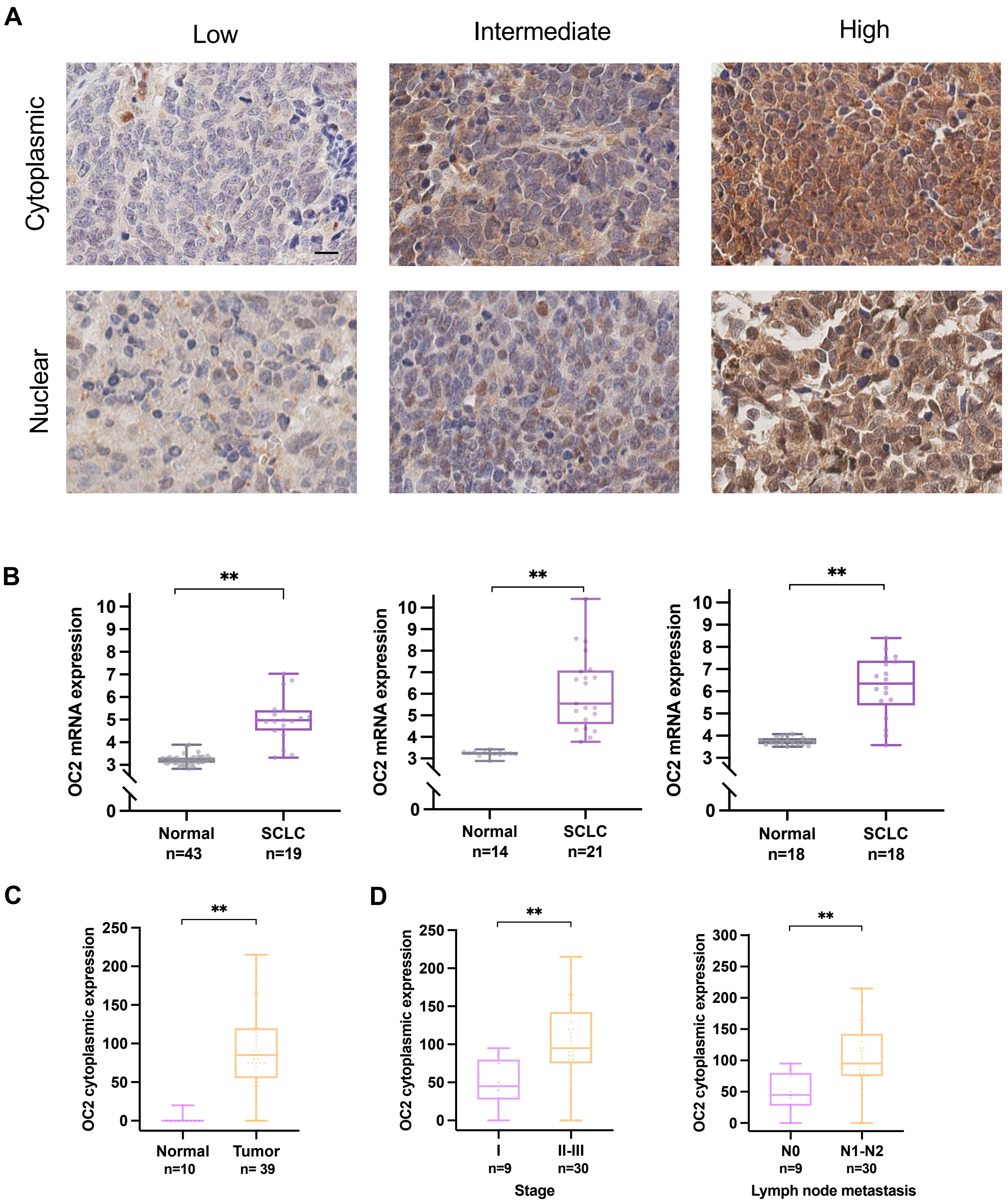

Supplementary Figure 1. A) Representative IHC images of cytoplasmic (upper panel) and nuclear (lower panel) OC2 expression levels. OC2, brown. Scale bar, 50µm. **B)** OC2 mRNA expression (RMA, Robust Multiarray Average) in normal lung tissue compared to SCLC from the GSE30219 (Rousseaux et al. 2013), GSE149507 (Cai et al. 2021), GSE40275 (Kastner et al. 2012) cohorts. The boxes show the 25-75th percentile range, and the center line is the median. Whiskers extend from the minimum and maximum values. For GSE30219 and GSE149507 cohorts, unpaired two-tailed Student´s t-test was performed. For the GSE40275 cohort, Wilcoxon two-tailed rank-sum test was performed. *=P<0.05, **=P<0.01. **C**) Quantification of cytoplasmic OC2 expression in benign and SCLC tissue. **D)** Association of stage (left) and lymph node metastasis (right) with OC2. Boxplots of intensity levels of cytoplasmic OC2 expression assessed by IHC using a SCLC TMA are shown. For **(C, D)** the boxes show the 25-75th percentile range, and the center line is the median. Whiskers extend from the minimum and maximum values. P-values were obtained from Wilcoxon two-tailed rank-sum test.

Supplementary Figure 2

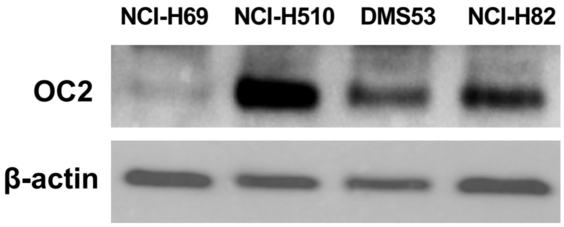

Supplementary Figure 2. Immunoblot showing endogenous OC2 levels in human SCLC cell lines.

Supplementary Figure 3

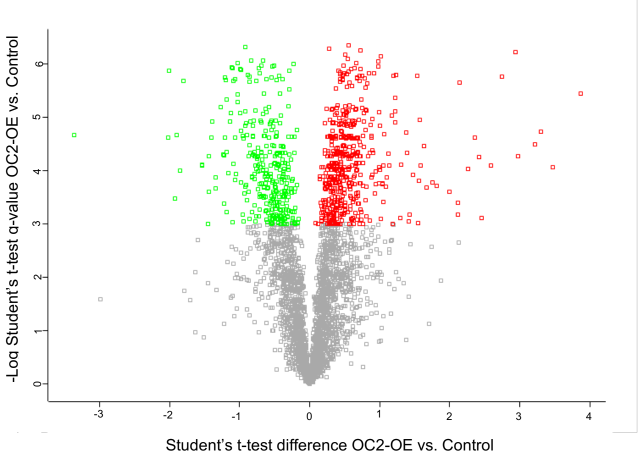

Supplementary Figure 3. Volcano plot showing DEPs (P-value<0.05) after OC2 induction in NCI-H510 cells.

Supplementary Figure 4

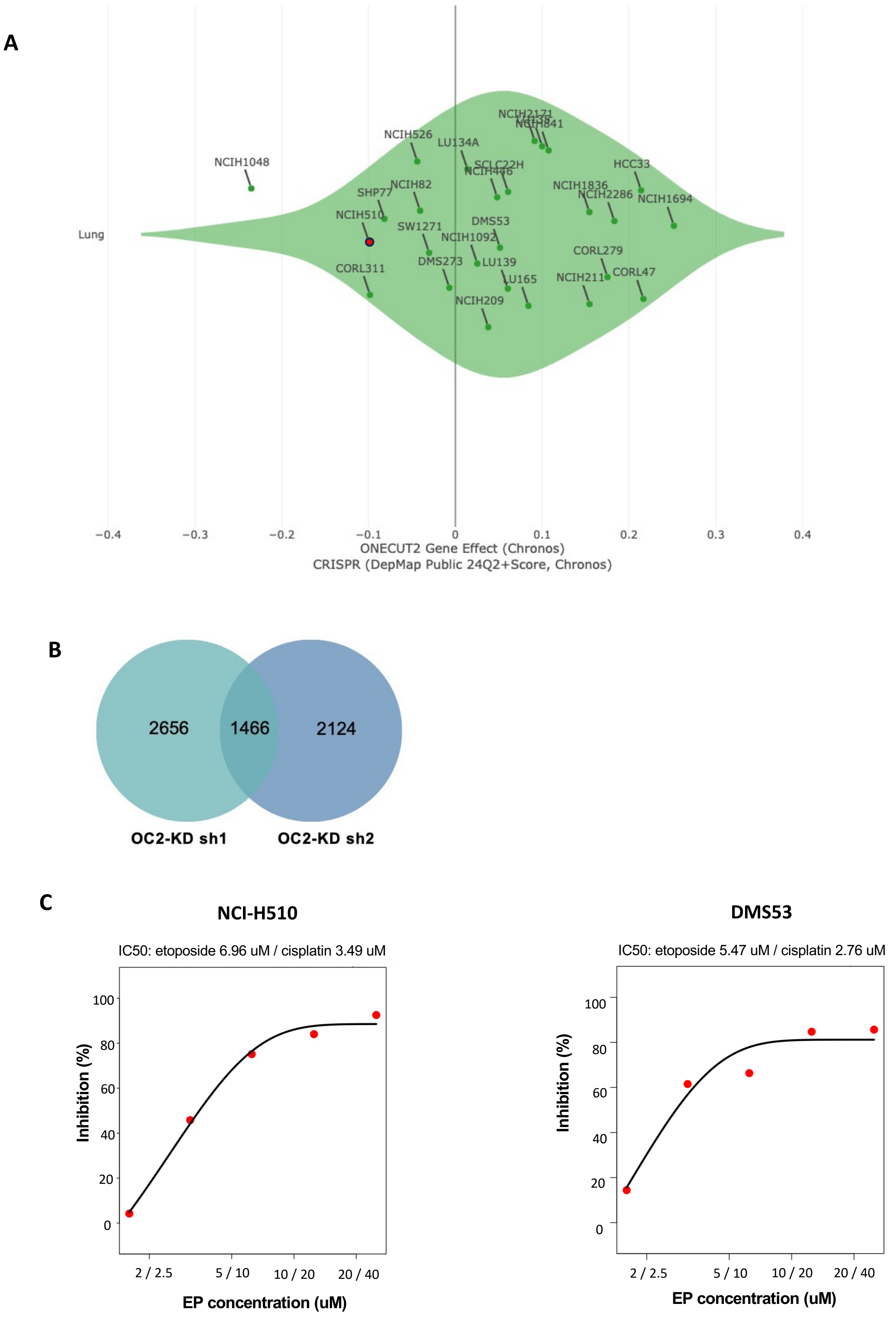

Supplementary Figure 4. A) CRISPR screening data showing OC2 dependency in the SCLC cell lines from DepMap (Therniak et al. 2017). **B)** Venn diagram of the overlapping DEGs after OC2 depletion with shRNAs in the NCI-H510 cell line. **C)** Dose-response curves and IC50 values for EP after 48 h treatment. Curves generated with SynergyFinder. The values shown are the mean ± S.D. from three independent experiments.

Supplementary Figure 5

A

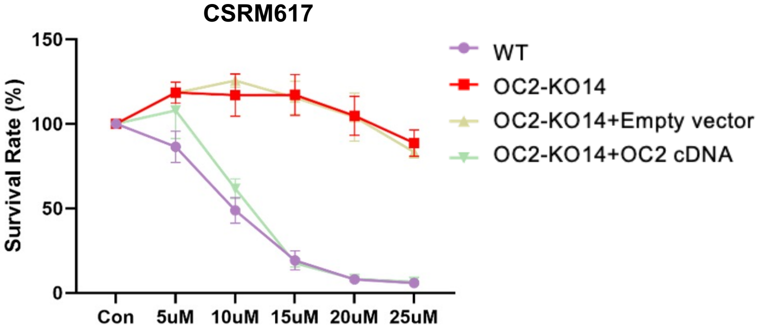

B

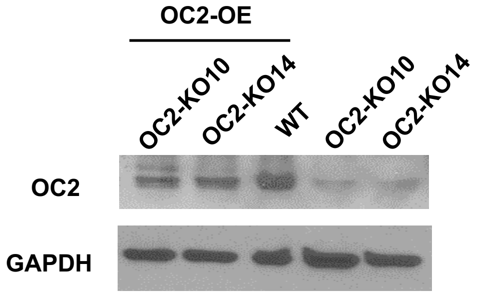

C

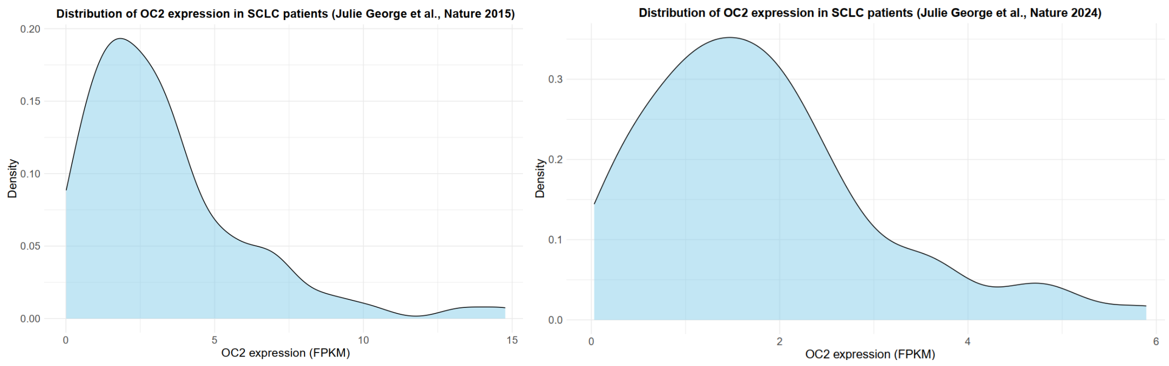

Supplementary Figure 5. OC2 silencing abrogates sensitivity to compound CSRM617 in 22Rv1 cells. A) Dose-response curves for CSRM617 in engineered the 22Rv1 castration-resistant prostate cancer cell line following 48 h treatment. Two CRISPR clones were generated: OC2-KO10 and OC2-KO14, with OC2-KO14 displaying more robust phenotypes and used for further analysis. OC2-KO cells were generated using CRISPR-Cas9 with sgRNA sequence "TCTGCCCAACTACGGTCCGC" and PAM "CGG" in the LentiCRISPR v2 vector (Addgene #52961), followed by puromycin selection (2 µg/mL) for single-cell clone isolation. For rescue experiments, OC2-KO cells were stably transduced with either an empty vector or OC2-OE lentivirus. Cells were seeded at a density of 2,000 cells/well. Data represent the mean ± S.E.M. from three independent experiments, assessed via XTT assay (CST #9095). **B)** Western blot analysis shows OC2 expression levels across wild-type, OC2-CRISPR (KO), and rescue lines (OC2-OE). **C)** Density of OC2 expression in two independent SCLC cohorts (George et al. 2015; George et al. 2024).

Supplementary Table 1

Supplementary Table 1. Altered SCLC-A subtype markers (Schwendenwein et al. 2021) in the DMS53 RNA-Seq dataset (OC2-OE *vs.* Control).

Gene	log(FC)	P-value	Adjusted P-value
BCL2	-2.161	1.63E-39	4.82E-37
DLL3	-0.634	8.80E-17	8.40E-16
IGFBP5	-5.033	5.06E-48	9.10E-45
NFIB	-0.445	1.08E-06	3.23E-06
RET	-2.511	3.38E-41	1.43E-38
TTF1	-0.167	0.023	0.037
SOX2	-0.974	1.39E-21	2.29E-20
SYP	-0.391	5.56E-18	6.16E-17

Supplementary Table 2

Supplementary Table 2. Altered SCLC-N subtype markers (Schwendenwein et al. 2021) in the DMS53 RNA-Seq dataset (OC2-OE *vs.* Control).

Gene	log(FC)	P-value	Adjusted P-value
ANTXR1	-1.003	2.73E-13	1.73E-12
AURKA	-1.726	9.17E-33	8.13E-31
HES6	-1.391	9.49E-29	4.77E-27
INSM1	-1.878	1.21E-36	1.94E-34
NCAM	-1.946	6.11E-34	6.64E-32
NFIB	-0.445	1.08E-06	3.23E-06

Supplementary Table 3

Supplementary Table 3. Altered SCLC-P subtype markers (Schwendenwein et al. 2021) in the NCI-H510 inducible system RNA-Seq dataset (OC2-OE *vs.* Control).

	First induction			Off-phase			Second induction		
**Gene**	**log(FC)**	**P-value**	**Adjusted P-value**	**log(FC)**	**P-value**	**Adjusted P-value**	**log(FC)**	**P-value**	**Adjusted P-value**
AVIL	4.325	4.78E-40	7.68E-37	0.523	2.04E-05	8.69E-04	4.355	4.94E-40	6.89E-37
MYC	0.839	8.29E-12	1.34E-10	0.462	0.001	0.022	1.366	7.99E-17	1.96E-15
CDH1	0.498	1.21E-21	8.06E-20	0.153	2.50E-06	1.49E-04	0.607	7.94E-25	7.01E-23
SOX9	1.215	4.54E-18	1.89E-16	-0.641	1.98E-07	1.64E-05	1.088	8.21E-16	1.76E-14
POU2F3	3.72	1.72E-05	9.36E-05	-0.003	0.997	0.999	4.717	1.61E-07	9.50E-07

Supplementary Table 4

Supplementary Table 4. Altered SCLC-A subtype markers (Schwendenwein et al. 2021) in the NCI-H510 inducible system proteomic dataset (OC2-OE *vs.* Control).

Protein	log(FC)	P-value
ASCL1	-0.298	1.27E-04
BCL2	-0.361	1.91E-04
IGFBP5	-1.013	1.09E-03
DLL3	-0.216	3.98E-03
